# A cross-sectional study of Health Related Quality of Life and body mass index in a Norwegian school sample (8–18 years): a comparison of child and parent perspectives

**DOI:** 10.1186/s12955-015-0239-z

**Published:** 2015-04-09

**Authors:** Sølvi Helseth, Kristin Haraldstad, Knut-Andreas Christophersen

**Affiliations:** Faculty of Health, Oslo and Akershus University College of Applied Sciences, P.O. box 4, St Olavs Plass, 0130 Oslo, Norway; Faculty of Health- and Sport Sciences, University of Agder, P.O. box 422, 4604 Kristiansand, Norway; Faculty of Social Sciences, University of Oslo, P.O. box 1084, Blindern, 0317 Oslo, Norway

**Keywords:** HrQoL, BMI, Children, Adolescents, Parents, KIDSCREEN

## Abstract

**Background:**

Because consequences of pediatric overweight and obesity are largely psychosocial, the aim of this study was to describe health related quality of life (HRQoL), the prevalence of overweight and obesity, and to examine the relationships between HRQoL and body mass index (BMI), age, and gender in a Norwegian sample of schoolchildren. In addition, because children are dependent upon their parents’ judgment of their condition, the aim was also to compare child- and parent-reported HRQoL and BMI, age, and gender.

**Methods:**

This cross-sectional study involved 1238 children (8–18 years) and 828 parents. HRQoL was measured with the Norwegian version of the KIDSCREEN-52, child and parent version. Child BMI was calculated based on objective measures of height and weight, and adjusted for age and gender. Multiple regressions were used to determine how variations in BMI, age, and gender affected child- and parent-reported HRQoL.

**Results:**

HRQoL decreased significantly with age and girls had lower HRQoL than boys on the majority of the KIDSCREEN subscales. Of the total sample, approximately 16% were overweight and 3% were obese. BMI contributed significantly to explaining the variations in the KIDSCREEN subscales of Physical well-being and Self-perception. Higher BMI was associated with lower HRQoL scores. Although there were significant differences between child and parent ratings on most KIDSCREEN subscales, the direction of the differences varied. In some scales, parents rated their child’s HRQoL higher than the child, and in some scales lower. Increasing age of the child seems to increase the differences, while gender and the child being overweight and/or obese affected the differences to a smaller extent.

**Conclusions:**

This study showed that almost 20% of the children and adolescents in a representative Norwegian school sample were overweight or obese. Age and gender were the most significant factors associated with variations in HRQoL in the sample; however, increasing BMI added to the negative effect of other factors. The study also found substantial differences between the child and parent ratings of the child’s HRQoL. Misinterpretations of the child’s well-being might result in less targeted actions to improve the child’s HRQoL.

## Background

Overweight and obesity are a serious public health problem, associated with various impairments and medical disorders [1-3]. The prevalence of overweight and obesity in childhood has increased rapidly during the last decade, although there is wide variation between countries. In the WHO-HBSC study, the results showed a prevalence above 10% among school-aged children in most nations, with a range of 7.6% (Latvia) to 28.8% (USA) [4]. There is some evidence that the rise has reached a plateau; nevertheless, the prevalence remains high across countries and is still rising in some countries [4-7]. Although overweight and obesity are not as strongly associated with morbidity in childhood, they are linked to low physical activity, underachievement in school, low self-esteem, social exclusion, and low quality of life [8-13]. Further, being overweight early in life is a strong predictor of obesity later in life [1,14,15].

Because consequences of pediatric obesity are largely psychosocial, the exploration of health related quality of life (HRQoL) seems relevant to understanding the effect of overweight and obesity on children and adolescents. HRQoL is generally conceptualized as a multidimensional construct that includes the individuals’ subjective perspective on physical, psychological, social, and functional aspects of health [16]. The multidimensionality of HRQoL measures provides researchers and clinicians with information about the impact of a health condition such as obesity, or the effect of various interventions on different aspects of quality of life, and serves as a framework for identifying and developing strategies to promote quality of life [17]. Previous studies have identified increased body mass index (BMI) as a significant factor associated with poor HRQoL in children and adolescents [10-12,18-22]. In a review of studies, an inverse relationship between BMI and pediatric HRQoL, with impairments in physical and social functioning, was consistently reported [11]. Because HRQoL in young populations varies systematically with age and gender [23], and little is known about how these characteristics may account for variations in impairment of HRQoL in overweight and obese children, research on HRQoL should consider these factors. In a German study, reduced HRQoL was found in three subscales; Physical well-being, Psychological well-being, and Self-perception, regardless of age and gender. The pattern of impairment in the dimensions of HRQoL was similar across ages, with children (8–11 years) having greater obesity-related impairment than adolescents (12–16 years); however, the absolute lowest scores were reported by adolescents in general, and specifically by adolescent girls [24]. In an Australian community sample study, HRQoL was significantly lower for overweight, and even lower for obese adolescents than for other adolescents. The Physical and Social functioning scales were most impaired, and obese girls reported lower HRQoL than obese boys [25]. This corresponds with research that shows that adolescents report lower QOL than children, and girls have lower QOL than boys [26]. To our knowledge, no studies have explored the relationship between HRQoL dimensions, BMI, age, and gender in a Norwegian community sample of children.

Emerging research suggests that treatment-seeking populations of overweight children have poorer quality of life than overweight children in community samples [11,24]. Knowing that children are dependent upon their parents or caregivers to enter them into a treatment program, one might speculate that overweight children are not referred to treatment before their well-being is seriously diminished. Because parent–child agreement on the child’s HRQoL in general is moderate to low and changes with the child’s increasing age [27-29], there is a risk that adolescents’ levels of well-being might be misinterpreted by parents and thus, low levels of HRQoL might not be acted upon. Clearly, more knowledge is needed on how parents estimate the quality of life of their overweight children, to be able to detect and act upon signs of decreasing HRQoL in overweight children at an early stage.

The aims of this study in a representative school sample of children (8–18 years) in Eastern Norway were as follows.

1. To describe HRQoL and the prevalence of overweight and obesity.
2. To examine the relationship between HRQoL and overweight/obesity, gender, and age.
3. To examine the relationship between child- and parent-reported HRQoL and overweight/obesity, gender, and age.

In addition, the psychometric properties of the parent version of the Norwegian KIDSCREEN-52 were evaluated.

## Methods

### Sampling

A cross-sectional study was conducted in an eastern region of Norway with about 1.7 million inhabitants (36% of the total Norwegian population), and a child population (8–18 years) of about 230,000. The sampling was planned and conducted in cooperation with Statistics Norway. First, all schools in the region were stratified according to population density, school size, and school level. We randomly selected two schools from each stratum, and non-responding schools were replaced by schools selected according to the same criteria. From the cluster sample of 20 schools, we selected classes that covered grades 3, 5, 7, and 9 in elementary schools, and grades 1 and 3 in secondary schools. We invited the children and adolescents to participate after their schools agreed to be part of the study. We recruited 1675 children and adolescents aged 8–18 years, and gave them and their teachers standard verbal and written information about the study one week before they participated. A signed informed consent was obtained from the parents, and a signed written informed assent was taken from the eligible children. Students completed the self-report instruments in their classrooms during school hours, and the investigator and the teacher were present to provide assistance when needed. For the grade 3 elementary schoolchildren, the investigator read the questionnaire aloud to the class. Those who were absent from school on the day of the study were not included. Of the 1675 eligible children and adolescents, 258 (15%) did not provide written permission from their parents, 148 (9%) were absent from school on the day of the study, and 31 (2%) did not want to participate. Hence, 1238 children and adolescents participated, for an overall response rate of 74%. The response rate varied across schools from 55% to 96%. In addition, 828 parents (67% of the responding children) answered the questionnaires. The study was reviewed and approved by the Regional Research Ethics Committee of Norway.

### Instruments

#### Demographic variables

The first part of the questionnaire recorded demographic details of nationality, gender, date of birth, cohabitant status, parental marital status, and school year. The parental marital status was dichotomized as two parents (married or cohabiting) or single parent (unmarried, divorced, or widowed).

#### Health related quality of life

The Norwegian version of the KIDSCREEN-52 was used to measure HRQoL. The KIDSCREEN questionnaire is a generic HRQoL instrument based on a multidimensional HRQoL construct [29]. The questionnaire focuses on physical, mental, and social dimensions of well-being, and it measures HRQoL from the perspective of the child or adolescent. The instrument is available in three versions: 52 items, 27 items, and 10 items, and has a child and a proxy version. The 52-item, child, and proxy (parent) versions were used in this study. The instrument includes 52 items, which are rated by each individual on a five-point Likert scale. The scale indicates either the frequency of certain behaviors or feelings (1 = never, 2 = seldom, 3 = sometimes, 4 = often, 5 = always) or the intensity of an attitude (1 = not at all, 2 = slightly, 3 = moderately, 4 = very, 5 = extremely). The time frame refers to the previous week. The 52 items are distributed into the following 10 aspects or dimensions: Physical well-being (five items), Psychological well-being (six items), Moods and emotions (seven items), Self-perception (five items), Autonomy (five items), Parent relations (six items), Social support and peers (six items), School environment (six items), Social acceptance/bullying (three items), and Financial resources (three items). The scale for negatively worded items was reversed and missing values were substituted by the mean of the nonmissing items; however, no score was computed if more than one item per scale was left unanswered. The three-item scales required that all items be filled in. The dimension score was then transformed linearly to a 0–100-point scale, with 100 indicating the best QOL and 0 the worst. The KIDSCREEN-52 has been translated into several languages and its cross-cultural comparability and psychometric properties have been found satisfactory in the different language versions [30-33]. The Norwegian child version has shown satisfactory validity and reliability [34]. The psychometric properties of the parent proxy version were tested as part of this study.

#### Body mass index

BMI was calculated for each individual by dividing their weight in kilograms by their height in square meters. Age- and gender-specific BMI cut-off values, proposed by the International Obesity Task Force, were used to categorize the adolescents as overweight or obese [35]. BMI < 25 is considered normal weight, 25 ≤ BMI < 30 is considered overweight, and BMI ≥ 30 is obese. Because self-reported body weight is often inaccurate [36], trained nurses measured the students’ height and weight in separate rooms while they were wearing street clothes but no shoes. We used a SECA 761 scale, which is EU approved and was developed for medical use. Standing height was measured with a Seca stadiometer.

### Analyses

The analyses were conducted using SPSS (20.0) and AMOS (20.0). Descriptive statistics for the variables of BMI, age, gender, and HRQoL are presented as means and SDs or as frequencies and percentages. Pearson’s correlations were calculated to examine the relationships between each of the 10 KIDSCREEN scales (child-reported) and the independent variables. After examining the bivariate relationships and considering whether schools differed on the dependent variable and whether clustering should therefore be taken into account, multiple regressions were used to determine how variations in BMI, age, and gender affected child-reported HRQoL. Finally, the data from the parent reports and the corresponding child reports were merged; with the differences between child and parent reports on each of the KIDSCREEN scales put into the models as the dependent variable, and regression was performed to detect differences in how parents and children report on children’s HRQoL when BMI, age, and gender vary.

Mean, standard deviations, measures of skewness and kurtosis, and floor and ceiling effects were calculated for each scale of the KIDSCREEN-52 proxy version. Reliablity is expressed by Cronbach’s alpha and was computed for the 10 KIDSCREEN scales. Alpha coefficient of .70 or above was considered acceptable. To test how well the KIDSCREEN-52 proxy version fit the data, confirmatory factor analysis (CFA) was conducted, using structural equation modeling (SEM) analysis. CFA models were tested for each of the 10 scales and for the complete KIDSCREEN measure using the sums of each scale. The latter analysis was conducted with sum indicators for all ten scales and for the first nine scales (excluding bullying). CFA provides goodness-of-fit tests and allows alternative models to be tested. Acceptable goodness-of-fit values indicate the construct validity of a model, and the literature contains various recommendations about the type, number, and cutoff values for goodness of fit that are required to be reported. The following goodness-of-fit indices were used in this study: chi-square (χ^2^), adjusted goodness-of-fit index (AGFI), Tucker–Lewis index (TLI), comparative fit index (CFI), and the root mean square error of approximation (RMSEA). Acceptable goodness of fit was defined as [37,38]: nonsignificant χ^2^ (sensitive to sample size); AGFI > 0.90 (acceptable) ≥ 0.98 (good); TLI > 0.90 (acceptable), ≥ 0.95 (good); CFI > 0.90 (acceptable) ≥ 0.95 (good); RMSEA < 0.05 (close fit) ≤ 0.08 (fair).

## Results

Table 1 shows the sample characteristics on HRQoL and BMI by age and gender. Age was grouped into three categories: 8–11, 12–15, and 16–18 years. BMI was grouped into normal weight, overweight, and obese. The sample size eligible for analysis was 1066 after excluding children with missing values. Girls were slightly overrepresented in the sample. Further, girls reported significantly lower HRQoL than boys on the KIDSCREEN subscales Physical well-being, Psychological well-being, Mood, Self-perception, Autonomy, and Social support and peers. On the subscales of Parent relations, Financial resources, School environment, and Bullying, there were no significant gender differences. Overall, HRQoL decreased significantly with age in both girls and boys on all subscales, except for Financial resources for girls and Mood for boys. Of the total sample, 80.7% were normal weight, 16.3% were overweight, and 3.0% were obese. However, girls and boys showed a different age-related pattern in overweight and obesity. While a larger proportion of the oldest girls (16–18 years) were overweight and/or obese than the younger girls (p = 0.03), a smaller proportion of the oldest boys were overweight and/or obese than the younger boys (n.s.). However, in total, there were no gender differences concerning overweight and obesity.

**Table 1:** **Health related quality of life and BMI by gender and age (n = 1066)**

		**Girls**					**Boys**					**Total**		
**KIDSCREEN scales**	**No of items**	**8–11**	**12–15**	**16–18**	**Total**	**p** ^**a**^	**8–11**	**12–15**	**16–18**	**Total**	**p** ^**a**^	**p** ^**b**^	**p** ^**c**^	**n = 1066**
		**n = (166)**	**n = (246)**	**n = (164)**	**n = 576**		**n = (134)**	**n = (216)**	**n = (140)**	**n = 490**				
Physical well-being	5	70.06	66.61	54.51	64.16	<.001	73.17	71.67	65.00	70.17	.001	<.001	<.001	66.92
Psychological well-being	6	83.16	77.24	69.16	76.64	<.001	83.61	80.54	73.75	79.44	<.001	.009	<.001	77.93
Mood	7	82.90	82.78	73.69	80.23	<.001	84.30	83.90	82.63	83.54	.541	<.001	<.001	81.80
Self-perception	5	82.86	68.16	56.28	69.03	<.001	86.87	81.57	74.00	80.86	<.001	<.001	<.001	74.47
Autonomy	5	74.91	72.68	56.01	68.58	<.001	75.97	75.09	67.21	73.08	<.001	<.001	<.001	70.65
Parent relations	6	82.56	77.41	70.12	76.82	<.001	83.58	78.28	75.12	78.83	<.001	.086	<.001	77.74
Financial resources	3	72.24	76.76	78.15	75.85	.053	71.52	79.63	79.52	77.38	.003	.289	<.001	76.56
Social support and peers	6	77.84	76.13	72.97	75.72	.041	76.06	72.59	68.78	72.45	.003	.003	<.001	74.22
School environment	6	79.44	67.06	57.72	67.97	<.001	76.15	66.30	54.70	65.68	<.001	.064	<.001	66.92
Bullying	3	87.05	90.14	95.58	90.80	<.001	86.26	90.51	93.15	90.21	.001	.468	<.001	90.48
BMI n (%)														
Normal		135(81.3)	210(85.4)	123(75)	468(81.2)	.032	105(78.4)	171(79.2)	116(82.9)	392(80)	.559	.783		860(80.7)
Overweight		28(16.9)	31(12.6)	31(18.9)	90(15.6)		27(20.1)	37(17.1)	20(14.3)	84(17.1)				174(16.3)
Obese		3 (1.8)	5 (2.0)	10(6.1)	18(3.1)		2(1.5)	8(3.7)	4(2.9)	14(4.6)				32(3.0)

p^a^ overall age differences within gender (f-test); p^b^ gender differences (t-test); p^c^ overall age differences (f-test).

### Relationships between HRQoL, age, gender, and BMI

The relationships between the variables were examined by calculating Pearson’s product moment correlation coefficients. In these analyses, the variables of age and HRQoL were considered continuous, while BMI and gender were dichotomous. BMI was grouped into normal weight (BMI < 25) and overweight and obese (BMI ≥ 25). As shown in Table 2, BMI was negatively and moderately to weakly correlated with the HRQoL subscales of Physical well-being and Self-perception, which indicates that children with higher BMI scored lower on Physical well-being and Self-perception. Negative and moderate correlations were also found between age and the HRQoL subscales of Physical well-being, Psychological well-being, Mood, Self-perception, Autonomy, Parent relations, School support and peers, and School environment. A weak positive association was found with Financial resources and Bullying. These results indicate that HRQoL decreases with age in almost all dimensions. Gender was weakly and positively correlated with four of the HRQoL subscales. A positive, moderate correlation was found in the Self-perception subscale, meaning boys have better self-perception than girls.

**Table 2:** **Bivariate correlations between HRQoL and gender, age, and overweight, and explained variance of gender, age and overweight on HRQoL (n = 1066)**

	**Bivariate correlations**			**Model 1**			**Model 2**		**Model 3**	
**Scales**	**Gender**	**Age**	**Overweight**	**R** ^**2**^	**Gender** ^**a**^	**Age** ^**a**^	**R** ^**2**^	**Interaction gender and age** ^**a**^	**R** ^**2**^	**Overweight** ^**a**^
Physical well-being	.15	–.24	–.14	0.080	0.022	0.058	0.085	0.005	0.099	0.019
Psychological well-being	.08	–.28		0.086	0.006	0.080	0.088		0.087	
Mood	.11	–.17		0.040	0.013	0.027	0.050	0.010	0.041	
Self-perception	.29	–.38	–.07	0.228	0.082	0.146	0.246	0.018	0.232	0.004
Autonomy	.11	–.29		0.094	0.012	0.082	0.103	0.009	0.096	
Parent relations		–.23		0.054		0.051	0.055		0.054	
Financial resources		.10		0.011		0.010	0.011		0.012	
Social support and peers	–.09	–.14		0.027	0.008	0.018	0.028		0.027	
School environment		–.42		0.182	0.003	0.179	0.182		0.183	
Bullying		.19		0.035		0.035	0.036		0.036	

Correlation between gender and age, *r* = .001, p = .995.

Only significant (p < .05) results are shown in the table.

^a^unique contribution.

Model 1: Gender and age with the unique contributions of gender and age in addition to total R^2^.

Model 2: Gender, age, and interaction between gender and age with the unique contribution of the interaction in addition to total R^2^.

Model 3: Gender, age, and overweight with the unique contribution of overweight in addition to total R^2^.

To examine further how BMI, age, and gender were associated with HRQoL, multiple linear regression analyses were performed with each KIDSCREEN subscale as dependent variables. Multilevel analysis was considered because data were collected at schools. However, we decided to use linear regression of three reasons. First, the grouping was weak for eight scales (.01 < ICC < .10) and only moderate for two of the scales (ICC ≥ .16). Second, the level 1 variables explained only a small amount of level 1 (1% to 9%), but a large part (52% to 91%) of the level 2 variance. This is because class and age are highly collinear and therefore a bit misleading. Third, our focus was on explained variance of HRQoL, and not on variation of regression coefficients.

The independent variables were entered stepwise into the model: gender, age, interaction between gender and age, and finally, BMI. Table 2 shows the significant results (p < .05) of total R^2^ and the unique contributions of each of the independent variables. In model 1, R^2^ varied between 0.011 and 0.228, meaning that gender and age accounted for 1–23% of the variance in the KIDSCREEN subscales. Age made the biggest contribution in all subscales. However, it is noticeable that gender explained 8% of the variance in the Self-perception subscale, which was the largest contribution of gender in this model. Adding interaction between gender and age increased the explained variance significantly, but to a small extent, in the subscales of Physical well-being, Mood, Self-perception, and Autonomy. Adding BMI resulted in negligible changes in the explained variance. However, BMI contributed significantly but weakly to explaining variations in the subscales of Physical well-being and Self-perception.

### Comparison of parent and child results

Reliability of the subscales in the parent version of the KIDSCREEN-52 was confirmed by acceptable levels of Cronbach’s alpha for all scales (Table 3). Table 3 shows the goodness-of-fit indices for the KIDSCREEN subscales and total scale. Two of the scales only had three items (Financial resources and Bullying) and CFA was not conducted for these scales. The RMSEA values ranged from 0.03 to 0.07, indicating close to acceptable fit for all scales. The AGFI indices showed acceptable to good fit (0.96–0.99), while the CFI indices were all above 0.97, which implies a good fit. Finally, the TLI indices were all above 0.95, implying a good fit. The CFA confirms that the sub-scales and total scale of the Norwegian proxy version of the KIDSCREEN-52 fit the data well. However, the χ^2^ was significant for all scales except Self-perception and Parent relations. This may be because of the relatively large sample.

**Table 3:** **Goodness-of-fit indices** ^**a**^ **and Cronbach’s alpha for the KIDSCREEN-52 proxy version (n = 639)**

**Scales**	**P (χ** ^**2**^ **)**	**RMSEA**	**AGFI**	**TLI**	**CFI**	**Alpha**
Physical well-being	.023	.054	.974	.984	.993	.82
Psychological well-being	.012	.052	.971	.989	.996	.89
Mood	.007	.057	.963	.964	.978	.82
Self-perception	.085	.041	.981	.988	.995	.77
Autonomy	.005	.066	.965	.971	.988	.80
Parent relations	.179	.027	.984	.995	.998	.84
Financial resources^b^						.86
Social support and peers	<.001	.074	.988	.975	.992	.88
School environment	.003	.060	.964	.980	.992	.85
Bullying^b^						.79
Kidscreen, 9 scales	<.001	.051	.959	.972	.981	.86
Kidscreen, 10 scales	<.001	.058	.945	.958	.970	.83

^a^Indices: chi-square (χ^2^), the root mean square error of approximation (RMSEA), goodness of fit index (AGFI), Tucker–Lewis index (TLI), and comparative fit index (CFI).

^b^Scales (Finance and Bullying) with only three items each were not included in the CFA.

To assess how parents rated their child’s HRQoL in relation to the child’s own estimates, parent proxy reports were compared with the children’s reports and the mean differences between them were calculated. Complete data on parent–child pairs were found for 639 cases. Each KIDSCREEN scale with more than three items were examined for measurement invariance. The measurement differences between parents and children were consistently small enough to compare parents and children. Overall, there are substantial significant differences between parents and children in most KIDSCREEN subscales (Table 4). However, the direction of the relationships varies. In the subscales of Physical well-being, Self-perception, Autonomy, Financial resources, and School environment, parents tended to judge the quality of life of their children as better than the children did, while in the subscales of Psychological well-being, Parent relations, and Social support and peers, parents rated their children’s quality of life as poorer then the children did. To examine further how the variables of gender, age, and BMI are associated with the differences in parent proxy and child reports, multiple linear regression was performed with differences in parent and child ratings of HRQoL as the dependent variable (Table 4). The variables were entered sequentially (gender, age, and BMI) into the regression. Table 4 shows that age, gender, and BMI contributed to only a small extent in explaining variation in the differences. Gender explained only a small amount of the variation in three subscales (Self-perception, Autonomy, and Financial resources), indicating that the child’s gender was not an important factor to consider when explaining the differences in ratings between parents and children. The child’s age contributed significantly more than gender, but still only accounted for up to 7% of the variance in the differences on five subscales.The biggest contribution of age was found in the subscales of Self-perception (5%) and Financial resources (7%). BMI contributed significantly but weakly to the variance of Physical well-being and Psychological well-being (1–2%), indicating that the children’s BMI did not substantially explain differences in parent and child judgments of child quality of life.

**Table 4:** **Mean differences between parent proxy and child reports on the KIDSCREEN-52, and the explained variance of gender, age, and overweight on the differences in HRQoL ratings between parent proxy and child (n = 639)**

	**Mean difference**	**Model 1**			**Model 2**		**Model 3**	
**Scale**		**R** ^**2**^	**Gender** ^**a**^	**Age** ^**a**^	**R** ^**2**^	**Interaction** ^**a**^	**R** ^**2**^	**Overweight** ^**a**^
Physical well-being	5.68							0.006
Psychological well-being	−2.99	0.023		0.018	0.025		0.038	0.015
Mood								
Self-perception	4.66	0.090	0.036	0.049	0.116	0.026	0.092	
Autonomy	2.46	0.043	0.008	0.032	0.058	0.015	0.044	
Parent relations	−2.79	0.019		0.012	0.019		0.022	
Financial resources	2.07	0.072	0.007	0.068	0.077		0.073	
Social support and peers	−7.44							
School environment	6.58	0.017		0.014	0.018		0.021	
Bullying								

Correlation between gender and age: *r* = .06, p = .132.

Only significant (p < .05) results are shown in the table.

^a^unique contribution.

Model 1: Gender and age with the unique contributions of gender and age in addition to total R^2^.

Model 2: Gender, age, and interaction between gender and age with the unique contribution of the interaction in addition to total R^2^.

Model 3: Gender, age, and overweight with the unique contribution of overweight in addition to total R^2^.

## Discussion

Consistent with previous findings [26,39], HRQoL in this Norwegian representative sample showed the same pattern of variation between gender and age. Girls in general reported a lower HRQoL than boys, and older children/adolescents generally experienced a lower HRQoL than younger children.

Nationwide data on BMI across age groups during childhood in Norway were not available. However, the Norwegian Growth Study (representative nationwide data on 8-year-old children) [7], the Bergen Growth Study (representative data from western Norway on children aged 2–19 years) [40], and several other, more limited Norwegian and Scandinavian studies present a prevalence of overweight and obesity to which the findings of this study can be related. The prevalence of overweight and obesity in recent Norwegian studies varies between 11.5% and 16.9% for overweight and 2.3% and 5.1% for obesity, in children aged 2–19 years [7,40,41]. The prevalence for the total sample in this study was near the top of this range with 16.7% overweight and 3.3% obese, higher than in the Bergen Growth Study, which had an overall prevalence of overweight and obesity at 13.8% [40], but still somewhat lower than other European figures [42]. In the present study, age- and gender-related differences in the prevalence of overweight and obesity were studied. Older girls were significantly more often overweight and obese than younger ones (25% vs 18.7%), while the opposite was the case among boys, although not significant (17.1% vs 21.6%). Overall, there were no significant gender differences, and this is consistent with other studies [40,43]. It might be assumed that gender differences do not play a major role in explaining childhood overweight and obesity [44]. However, earlier research tends to show that at the youngest ages, girls are more often overweight or obese than boys, but at the higher ages this relationship is reversed [44]. In the Bergen Growth Study (aged 2–19 years), overweight and obesity were more prevalent in girls than in boys in the youngest age groups and more prevalent in boys in the oldest age group, but this leveled out in the total sample [40]. The Norwegian Growth Study (aged 8 years) and a study from Northern Norway (aged 6 years) also found a larger proportion of overweight and obesity among young girls than boys [41]. Our findings show a different pattern than other studies, with adolescent girls (16–18 years) more at risk of being overweight and obese than younger girls. Whether this is a sign of a new trend or due to methodological issues needs further study. However, this study covers a wider age range, has fewer participants in each age group, and was carried out in the most population-dense part of Norway (eastern Norway), which are all factors necessary to consider when comparing the findings with other studies. Urban–rural differences and social inequalities in the prevalence of overweight and obesity with gender interactions have been found and might be part of the explanation of the findings of this study [7,43,45].

BMI was not found to be a strong explanatory factor for variations in HRQoL in this study. However, findings from other studies are confirmed, with an inverse relationship between BMI and HRQoL in the subscales of Physical well-being and Self-perception [17,19,24]. The strongest associations between BMI and HRQoL have been found in obese children and adolescents, especially in treatment-seeking obese children [12,19,24,46], and the proportion of obese children in our study is very limited. This study, like most of the literature, shows that HRQoL varies between genders on the majority of subscales, meaning girls have poorer HRQoL than boys. However, the greatest variations in HRQoL are seen between different ages, and age contributes significantly to explaining the variations in all the HRQoL subscales. Overall, increasing age is associated with decreasing HRQoL, consistent with most previous findings [24,39]. Our findings emphasize the vulnerability of adolescents, especially adolescent girls, regardless of their BMI. However, BMI is negatively associated with the HRQoL subscales of Self-perception and Physical well-being, and it is therefore reasonable to assume that this will add to an already decreasing HRQoL in this vulnerable group.

The Norwegian parent proxy version of the KIDSCREEN-52, used here to study the effect of age, gender, and BMI on differences in parent–child reports on HRQoL, appears to be a valid and reliable measure, supporting the findings of a number of studies [33]. In the past, differences between child and parent reports have often been explained as methodological error and limited attention has been given to the clinical meaningfulness of such differences [47]. Consistent with other studies, we found significant differences between child and parent ratings in the majority of the KIDSCREEN subscales [47-49]. The direction of the differences varied on the different dimensions of HRQoL; in some domains, parents would overestimate, and in others underestimate their child’s self-reported HRQoL. Other studies have reported different directions of disagreement in different populations and within the same population, but studies of the directions within subdimensions have rarely been reported [47-50]. Such differences within subdimensions might affect the interpretation of differences in global scores of HRQoL, which is dominant in the literature [49]. Further, many studies have shown that the levels of parent–child agreement on dimensions of HRQoL vary and that the highest agreements are usually found for dimensions related to physical and cognitive functioning with lower agreement on emotional and social functioning [47,48]. This is only partly the case in our study, where the largest mean differences are found in School environment, Social support and peers, and Physical well-being. Sattoe et al. [49] defined threshold values for disagreement on the KIDSCREEN 10 as minor, intermediate, and major. On this scale, the mean differences between children and parents in our study would be minor or even below minor and, as Sattoe et al. [49] suggest, the disagreement might not be as meaningful as is often assumed in the literature. Eiser and Varni [47] conclude that we need to establish thresholds for when differences in HRQoL ratings are sufficiently large to be of concern in child health care. Thus, there is a need to determine the minimally important difference in parent–child ratings, according to both size and direction of discrepancies. Our study was conducted in a population covering a wide age range, with mostly healthy children and adolescents. In the literature, the level of agreement is found to differ in different age groups, and between clinical groups and normal populations, usually with a higher level of agreement in different clinical groups; furthermore, the parents of children with chronic conditions tend to underestimate their children’s HRQoL [47,48]. Parents of obese youths have also been found to perceive their children’s HRQoL as worse than the adolescents do themselves [11]. In our study, however, this was not evident. BMI significantly but weakly explained variations only in the subscales of Physical well-being and Psychological well-being between children and parents, which again should be interpreted in relation to the small subsample of obese children and adolescents in the study. However, it is reasonable to assume that higher BMI might lead to more disagreement in ratings. The most noticeable factor to significantly explain variations in the differences between parents and children in our study was age, indicating that the level of agreement lessened with increasing age. Gender did not make a considerable impact in our study.

## Conclusions

This study showed that almost 20% of the children and adolescents in a representative Norwegian school sample were overweight or obese. Age and gender were the most significant factors to consider regarding variations in HRQoL; however, increasing BMI added to the negative effect of other factors, and thus must be taken seriously when children’s HRQoL is evaluated. The study also shows that there are substantial differences between the ratings of children and parents of the children’s HRQoL. Misinterpretations of children’s well-being might result in less targeted actions to improve children’s HRQoL.
